# Modeling financial interval time series

**DOI:** 10.1371/journal.pone.0211709

**Published:** 2019-02-14

**Authors:** Liang-Ching Lin, Li-Hsien Sun

**Affiliations:** 1 Department of Statistics, National Cheng Kung University, Tainan, Taiwan; 2 The Graduate Institute of Statistics, National Central University, Taoyuan, Taiwan; Feng Chia University, TAIWAN

## Abstract

In financial economics, a large number of models are developed based on the daily closing price. When using only the daily closing price to model the time series, we may discard valuable intra-daily information, such as maximum and minimum prices. In this study, we propose an interval time series model, including the daily maximum, minimum, and closing prices, and then apply the proposed model to forecast the entire interval. The likelihood function and the corresponding maximum likelihood estimates (MLEs) are obtained by stochastic differential equation and the Girsanov theorem. To capture the heteroscedasticity of volatility, we consider a stochastic volatility model. The efficiency of the proposed estimators is illustrated by a simulation study. Finally, based on real data for S&P 500 index, the proposed method outperforms several alternatives in terms of the accurate forecast.

## Introduction

There are a large number of models to develop in order to analyze financial data. Conventionally, most of well-proposed models are constructed by daily closing price. By doing so, some important valuable intra-daily information may be discarded such as maximum and minimum prices. According to the recent literature, we can treat the maximum and minimum prices as an interval valued observations. Symbolic data methodologies are applied to deal with this approach. For instance, Billard and Diday [1, 2] propose the evaluation of mean, variance, and covariance along with regression analysis based on interval valued observations. By integrating the time dependency factor, their method evolves into the analysis of interval time series. The recent research pays more attention to model and forecast the interval time series process. In this study, we propose an interval time series model, and apply the proposed model to forecast the consecutive interval.

A naïve method to approach the interval time series is considering the maximum and minimum processes as a vector. This leads to the vector autoregressive (VAR) model. However, uncontrollable noise terms can bring about larger predicted lower value than the upper value. To deal with this problem, one can change the interval time series process to a bivariate time series model based on the center and the radius. For example, Neto and Carvalho [3] fit the autoregressive models to the center and radius processes, separately. It is possible to ignore the correlation between the center and radius. Arroyo et al. [4] consider their VAR model based on the first order difference center process and the radius process. Similarly, Rodrigues and Salish [5] introduce the centered returns, which is the difference between the current interval and the center value of the previous interval. They propose the center-radius self-exciting threshold autoregressive (CR-SETAR) model. Some related researches for interval time series include Gonzalez-Rodriguez et al. [6], Blanco et al. [7], and Gonzalez-Rivera et al. [8]. However, the parameters in the above models are estimated by the traditional least square estimators. The strictly positive radius may violate the normal assumption for the innovation terms. Besides, the parameters in the above models may not have an intuitive understanding since the structure of the intervals is already destroyed. Alternatively, Teles and Brito [9] propose the space-time autoregressive (STAR) model. By constraining the parameters, STAR can ensure that the predicted maximum value will be larger than the minimum value. However, we find by simulation that the phenomenon of the lower value to be larger than the upper value may happen when generating the interval observations.

In addition, Chou [10, 11] and Chen et al. [12] estimated the dynamic volatilities by using the ranges (the difference between the logarithmic maximum and minimum prices). For the former, based on the assumption of the asset to be driven by a geometric Brownain motion with stochastic volatility, Chou [10, 11] considered that the range as well as the upward and downward ranges (the difference between the logarithmic maximum/minimum and opening prices) follow a GARCH model. The parameters are obtained by quasi-maximum likelihood estimation in which the innovation term is given by an exponential distribution with the unit mean. For the latter, Chen et al. [12] further considered a threshold heteroskedastic model for the high/low ranges of asset prices. The innovation term is assumed to be a Weibull distribution.

In this study, we propose a model for financial interval time series. Instead of following the practice in the literature, we regard the process as a continuous path, where all observations are unobservable, except for the opening, maximum, minimum, and closing prices. According to this notion, the continuous path can be treated as high frequency data. Referring to Andersen et al. [13] and Aït-Sahalia et al. [14], we adopt the stochastic differential equation to characterize this continuous path. In order to construct the likelihood function of the maximum and terminal values (if the process is a standard Brownian motion), the reflection principle and the Girsanov theorem in Shreve [15] provide a technique. We can derive the conditional likelihood function in an analogous way. The advantages of our approach are: 1. the predicting/fitting maximum values are always larger than the predicting/fitting minimum values; 2. no constraint on the parameters is required to ensure the predicted maximum value to be larger than the minimum value; 3. the assumption of a strictly positive process can be avoided, since we do not transfer the observations to a (positive) radius process. Based on the proposed likelihood function, we obtain estimations of the parameters, and predict the one-step maximum and minimum values. Compared to Chou [10, 11] and Chen et al. [12], we derive the exact joint distribution for the maximum, minimum, and closing prices. Therefore, we can obtain the more accurate parameter estimations. To capture the heteroscedasticity in volatility, we also consider a stochastic volatility model. In particular, the volatility follows a daily stochastic differential equation where the marginal distribution of the volatility is inverse gamma distributed. From the simulations, we found that our estimation is more efficient than conventional sample covariance in the case of constant volatility, and the estimator proposed by Chou [10, 11] in the stochastic volatility model in terms of the relative error (RE). This advantage is likely due to using whole observable information instead of the closing price only. We also compare our approach with the frequently used alternatives to demonstrate its advantages.

## Main results

Referring to Andersen et al. [13] and Aït-Sahalia et al. [14], the intra-daily log price, a.k.a. the high frequency data, on the *i*-th day follows the stochastic differential equation

$$\begin{array}{c} dY_{t}=\mu dt+\sigma dW_{t},\;i-1<t<i, \end{array}$$ (1)

where *W*_*t*_ is a standard Brownian motion. In this study, we assume that all high frequency data are latent, except for the opening, maximum, minimum, and closing prices. Denote *X*_*i*_ = (*O*_*i*_, *U*_*i*_, *L*_*i*_, *C*_*i*_) as the observed random vector on the *i*-th day where *O*_*i*_, *U*_*i*_, *L*_*i*_, and *C*_*i*_ are the log opening, maximum, minimum, and closing price, respectively. The log maximum and minimum values can be given by *U*_*i*_ = max_*i*−1<*t*<*i*_ *Y*_*t*_ and *L*_*i*_ = min_*i*−1<*t*<*i*_ *Y*_*t*_. Applying the Girsanov theorem to *Y*_*t*_ and the connection between the maximum and the closing price expressed by Theorem 3.7.3 of Shreve [15], we have the following result.

**Theorem 1** *Suppose that the log price Y_t_ satisfies the stochastic differential* Eq (1), *and let* $O\overset{d}{=}O_{i}=Y_{i-1}$, $C\overset{d}{=}C_{i}=Y_{i}$ *and* $U\overset{d}{=}U_{i}=\max_{i-1\leq t\leq i}Y_{t}$. *Then the joint density of* (*U*, *C*) *conditional on O* = *o is*

$$\begin{array}{c} f_{U,C|O}\left(u,c|o\right)=\frac{2(2u-o-c)}{\sqrt{2\pi }\sigma^{3}}\;\text{exp}\;\{-\frac{{\left(2u-o-c\right)}^{2}}{2\sigma^{2}}-\frac{\mu^{2}}{2\sigma^{2}}+\frac{\mu (c-o)}{\sigma^{2}}\},u\geq o,\;u\geq c. \end{array}$$ (2)

Analogously, we have the following probability density function of the minimum and the closing log prices. Similarly, we can obtain the joint distribution of the terminal and the minimum values.

**Theorem 2** *Suppose that the log price Y_t_ satisfies* (1), *and let* $O\overset{d}{=}O_{i}=Y_{i-1}$, $C\overset{d}{=}C_{i}=Y_{i}$, *and* $L\overset{d}{=}L_{i}=\min_{i-1\leq t\leq i}Y_{t}$. *Then the joint density of* (*L*, *C*) *conditional on O* = *o is*

$$\begin{array}{ccc} f_{L,C|O}\left(\ell ,c|o\right) & = & \frac{2(o+c-2\ell )}{\sqrt{2\pi }\sigma^{3}}\text{exp}\{-\frac{{\left(o+c-2\ell \right)}^{2}}{2\sigma^{2}}-\frac{\mu^{2}}{2\sigma^{2}}+\frac{\mu (c-o)}{\sigma^{2}}\},o\geq \ell ,\;c\geq \ell . \end{array}$$

In addition, according to Choi and Roh [16], denoting $W_{t}^{u}=\sup_{0\leq s\leq t}W_{s}$ and $W_{t}^{l}=\inf_{0\leq s\leq t}W_{s}$, the joint distribution of $\left(W_{t},W_{t}^{u},W_{t}^{l}\right)$ is given by

$$\begin{array}{ccc} & & {I\;P}_{W_{t},W_{t}^{u},W_{t}^{l}}(a\leq W_{t}^{l}\leq W_{t}^{u}\leq b,W_{t}\in dx)\\ & = & \frac{1}{\sqrt{2\pi t}}\sum_{k=-\infty }^{\infty }[\text{exp}(-\frac{1}{2t}{\left(x-2k\left(b-a\right)\right)}^{2})\;\text{exp}\;(-\frac{1}{2t}{\left(x-2b-2k\left(b-a\right)\right)}^{2})]dx, \end{array}$$ (3)

with *a* ≤ 0 and *b* ≥ 0. Applying the Girsanov theorem, we obtain the joint density of the maximum, minimum, and closing log prices in the following theorem.

**Theorem 3** *Assume that the log price Y_t_ satisfies* (1), *and the conditions of Theorem 1 and Theorem 2 hold*. *Then the joint density of* (*U*, *L*, *C*) *conditional on O* = *o is*

$$\begin{array}{ccc} f_{U,L,C|O}\left(u,\ell ,c|o\right) & = & \sum_{k=-\infty }^{\infty }\frac{4k(k+1)}{\sqrt{2\pi }\sigma^{3}}(1-\frac{{\left(c+o-2u-2k\left(u-\ell \right)\right)}^{2}}{\sigma^{2}})f_{1}\\ & & -\sum_{k=-\infty }^{\infty }\frac{4k^{2}}{\sqrt{2\pi }\sigma^{3}}(1-\frac{{\left(c-o-2k\left(u-\ell \right)\right)}^{2}}{\sigma^{2}})f_{2}, \end{array}$$

*where ℓ* ≤ *c*, *o* ≤ *u and*

$$\begin{array}{ccc} f_{1} & = & \text{exp}\{-\frac{{\left(c+o-2u-2k\left(u-\ell \right)\right)}^{2}}{2\sigma^{2}}-\frac{\mu^{2}}{2\sigma^{2}}+\frac{\mu (c-o)}{\sigma^{2}}\},\\ f_{2} & = & \text{exp}\{-\frac{{\left(c-o-2k\left(u-\ell \right)\right)}^{2}}{2\sigma^{2}}-\frac{\mu^{2}}{2\sigma^{2}}+\frac{\mu (c-o)}{\sigma^{2}}\}. \end{array}$$

According to the results from Theorem 1 and Theorem 2, we can obtain the maximum likelihood estimators (MLEs) for the drift term *μ* and for volatility *σ*^2^ as follows.

**Proposition 1** *Suppose that the conditions of Theorem 1 and Theorem 2*. *Let X_i_* = (*O*_*i*_, *U*_*i*_, *L*_*i*_, *C*_*i*_) *for i* = 1, ⋯, *n be the observed data on the i-th day for the realization Y*. *The likelihood function of* (*μ*, *σ*^2^) *based on Theorem 1 is given by*

$$\begin{array}{c} L\left(\mu ,\sigma^{2}|\vec{u},\vec{o},\vec{c}\right)=\prod_{i=1}^{n}\frac{2(2u_{i}-o_{i}-c_{i})}{\sqrt{2\pi }\sigma^{3}}\;\text{exp}\;\{-\frac{{\left(2u_{i}-o_{i}-c_{i}\right)}^{2}}{2\sigma^{2}}-\frac{\mu^{2}}{2\sigma^{2}}+\frac{\mu (c_{i}-o_{i})}{\sigma^{2}}\},\;\;\; \end{array}$$

*where* $\vec{u}=\left(u_{1},\cdots ,u_{n}\right)$, $\vec{o}=\left(o_{1},\cdots ,o_{n}\right)$, *and* $\vec{c}=\left(c_{1},\cdots ,c_{n}\right).$ *Then the MLEs of μ and σ*^2^ *are*

$$\begin{array}{c} {\hat{\mu }}_{u}=\frac{1}{n}\sum_{i=1}^{n}\left(C_{i}-O_{i}\right),\\ {\hat{\sigma }}_{u}^{2}=\frac{1}{3n}\sum_{i=1}^{n}{\left(2U_{i}-C_{i}-O_{i}\right)}^{2}-\frac{1}{3}{\hat{\mu }}_{u}^{2}. \end{array}$$

*Similarly, using Theorem 2, the MLEs of μ and σ*^2^ *are*

$$\begin{array}{c} {\hat{\mu }}_{l}=\frac{1}{n}\sum_{i=1}^{n}\left(C_{i}-O_{i}\right),\\ {\hat{\sigma }}_{l}^{2}=\frac{1}{3n}\sum_{i=1}^{n}{\left(C_{i}+O_{i}-2L_{i}\right)}^{2}-\frac{1}{3}{\hat{\mu }}_{l}^{2}. \end{array}$$

Owing to ${\hat{\mu }}_{u}={\hat{\mu }}_{l}$, for simplicity, we use the notation $\hat{\mu }$ for both ${\hat{\mu }}_{u}$ and ${\hat{\mu }}_{l}$.

**Remark 1** *According to Theorem 3, the likelihood function can be written as*

$$\begin{array}{ccc} L(\mu ,\sigma^{2}|\vec{u},\vec{l},\vec{o},\vec{c}) & = & \prod_{i=1}^{n}\{\sum_{k=-\infty }^{\infty }\frac{4k(k+1)}{\sqrt{2\pi }\sigma^{3}}(1-\frac{{\left(c_{i}+o_{i}-2u_{i}-2k\left(u_{i}-\ell_{i}\right)\right)}^{2}}{\sigma^{2}})f_{1}\\ & & -\sum_{k=-\infty }^{\infty }\frac{4k^{2}}{\sqrt{2\pi }\sigma^{3}}(1-\frac{{\left(c_{i}-o_{i}-2k\left(u_{i}-\ell_{i}\right)\right)}^{2}}{\sigma^{2}})f_{2}\}, \end{array}$$

*with*

$$\begin{array}{ccc} f_{1} & = & \text{exp}\;\{-\frac{{\left(c_{i}+o_{i}-2u_{i}-2k\left(u_{i}-\ell_{i}\right)\right)}^{2}}{2\sigma^{2}}-\frac{\mu^{2}}{2\sigma^{2}}+\frac{\mu (c_{i}-o_{i})}{\sigma^{2}}\},\\ f_{2} & = & \text{exp}\;\{-\frac{{\left(c_{i}-o_{i}-2k\left(u_{i}-\ell_{i}\right)\right)}^{2}}{2\sigma^{2}}-\frac{\mu^{2}}{2\sigma^{2}}+\frac{\mu (c_{i}-o_{i})}{\sigma^{2}}\}, \end{array}$$

*where* $\vec{u}=\left(u_{1},\cdots ,u_{n}\right)$, $\vec{l}=\left(l_{1},\cdots ,l_{n}\right)$, $\vec{o}=\left(o_{1},\cdots ,o_{n}\right)$, *and* $\vec{c}=\left(c_{1},\cdots ,c_{n}\right).$ *The MLEs of μ and σ*^2^ *denoted as* $\left({\hat{\mu }}_{all},{\hat{\sigma }}_{all}^{2}\right)$ *can be obtained numerically*.

We then calculate the one-step predictions for the log maximum and minimum prices.

**Proposition 2** *From Proposition 1 and Remark 1, the one-step forecast of log maximum and minimum values are*

$$\begin{array}{ccc} U_{t}\left(1\right)=E\left[U_{t+1}|X_{1},\ldots ,X_{t}\right] & = & \sqrt{\frac{2{\hat{\sigma }}^{2}}{\pi }}\;\text{exp}\;\{-\frac{{\hat{\mu }}^{2}}{2{\hat{\sigma }}^{2}}\}+O_{t+1}+\hat{\mu }\Phi (\frac{\hat{\mu }}{\hat{\sigma }})\\ & & -\sqrt{\frac{{\hat{\sigma }}^{2}}{2\pi }}\;\text{exp}\;\left\{-\frac{{\hat{\mu }}^{2}}{2{\hat{\sigma }}^{2}}\right\}-\frac{{\hat{\sigma }}^{2}}{2\hat{\mu }}[1-2\Phi (\frac{\hat{\mu }}{\hat{\sigma }})],\\ L_{t}\left(1\right)=E\left[L_{t+1}|X_{1},\ldots ,X_{t}\right] & = & -\sqrt{\frac{2{\hat{\sigma }}^{2}}{\pi }}\;\text{exp}\;\left\{-\frac{{\hat{\mu }}^{2}}{2{\hat{\sigma }}^{2}}\right\}+O_{t+1}+\hat{\mu }\Phi (-\frac{\hat{\mu }}{\hat{\sigma }})\\ & & +\sqrt{\frac{{\hat{\sigma }}^{2}}{2\pi }}\;\text{exp}\;\left\{-\frac{{\hat{\mu }}^{2}}{2{\hat{\sigma }}^{2}}\right\}-\frac{{\hat{\sigma }}^{2}}{2\hat{\mu }}[1-2\Phi (-\frac{\hat{\mu }}{\hat{\sigma }})], \end{array}$$

*where* $\hat{\mu }$ *and* ${\hat{\sigma }}^{2}$ *are the MLEs based on X*_1_, …, *X*_*t*_.

Note that from Proposition 1 and Remark 1, we have the candidates for the MLE for *μ* (written as $\hat{\mu }$ and ${\hat{\mu }}_{all}$), and the MLE for *σ*^2^ (written as ${\hat{\sigma }}_{l}^{2}$, ${\hat{\sigma }}_{u}^{2}$, and ${\hat{\sigma }}_{all}^{2}$); see also the further discussion in Section: Simulations. Note that the quantity *O*_*t*+1_ can be set to *C*_*t*_ or it can be known. This means that we can make any decision after *O*_*t*+1_ is revealed.

In real-life applications, it is reasonable to assume that the mean return of each day equals zero. Then, we can obtain a simplified form for the one-step prediction.

**Corollary 1** *Let the assumptions of Proposition 1 hold, and further assume that μ* = 0. *Then we have*

$$\begin{array}{ccc} U_{t}\left(1\right)=E\left[U_{t+1}|X_{1},\ldots ,X_{t}\right] & = & O_{t+1}+\sqrt{\frac{2{\hat{\sigma }}^{2}}{\pi }},\\ L_{t}\left(1\right)=E\left[L_{t+1}|X_{1},\ldots ,X_{t}\right] & = & O_{t+1}-\sqrt{\frac{2{\hat{\sigma }}^{2}}{\pi }}. \end{array}$$

## Stochastic volatility model

A stochastic volatility model is constructed that the logarithmic price follows a stochastic diffusion equation and the volatility satisfies another diffusion processes. See, for instance, Hull and White [17], Stein and Stein [18], and Heston [19]. Define the stochastic volatility model as following:

$$\begin{array}{c} \left\{ \begin{array}{cccc} dY_{t} & = & \mu dt+\sigma_{t}dW_{t}, & Y_{0}=O,\\ d\sigma_{t}^{2} & = & b\left(\sigma_{t}^{2}\right)dt+\sqrt{v(\sigma_{t}^{2})}dB_{t}, & \sigma_{0}^{2}=\zeta , \end{array}\right. \end{array}$$

where (*B*_*t*_, *W*_*t*_)_*t*>0_ is a two-dimensional standard Brownian motion, and *O* is the initial log price and *ζ* is a random variable from the stationary distribution of $\sigma_{t}^{2}$ and independent of (*B*_*t*_, *W*_*t*_). Referred to Bibby et al. [20], we assume the drift function *b*(⋅) to satisfy the mean reverting function, that is, $b\left(\sigma_{t}^{2}\right)=\rho \left(\theta -\sigma_{t}^{2}\right)$. Then, the non-negative diffusion function *v*(⋅) is uniquely specified by the invariant density of $\sigma_{t}^{2}$. For example, if *v*(*x*) is proportion to a constant, *x*, or *x*^2^, the invariant density of $\sigma_{t}^{2}$ is respectively normal, gamma, or inverse gamma distributions. However, if the intra-daily volatility is a stochastic processes, the Girsanov theorem can not be applied straightforwardly. In this section, we consider that $\sigma_{t}^{2}$ is stochastic on the discrete time *i* = 1, 2, …, *n*, but has a stationary distribution during a fixed time interval *t* ∈ [*i* − 1, *i*].

To illustrate, we study a particular model. Referred to Bibby et al. [20], for *i* = 1, ⋯, *n*, the volatility $\sigma_{t}^{2}$ satisfies the following diffusion processes

$$\begin{array}{c} \sigma_{i}^{2}=\sigma_{i-1}^{2}+\rho (\frac{\beta }{\alpha -1}-\sigma_{i-1}^{2})+\frac{2\rho \sigma_{i-1}^{2}}{\alpha -1}{\tilde{Z}}_{i},\;\sigma_{0}^{2}=\zeta , \end{array}$$ (4)

where ${\tilde{Z}}_{i}$, *i* = 1, ⋯, *n*, are the standard normal random variables. By Bibby et al. [20], the stationary distribution of $\sigma_{i}^{2}$ is inverse gamma distributed. Then, given the *i*-th day volatility $\sigma_{i}^{2}$, the intra-daily log price *Y*_*t*_ on *i*-th day satisfies the following stochastic volatility model,

$$\begin{array}{c} Y_{i+j\Delta }=Y_{i+(j-1)\Delta }+\mu \Delta +\sigma_{i}Z_{j},\;j=1,\cdots ,m, \end{array}$$ (5)

where *Z*_*j*_, *j* = 1, ⋯, *m*, are independently and normally distributed with mean zero and standard deviation Δ = *m*^−1^. Namely, we assume that there are *m* log prices per day. For simplicity, we further assume that *Z* and $\tilde{Z}$ are mutually independent. Then, the joint density of (*U*, *C*, *L*) can be obtained by using Bayesian method as below

$$f_{U,L,C|O}\left(u,\ell ,c|o\right)=\int f_{U,L,C|O}\left(u,\ell ,c|o,s\right)f_{\sigma }\left(s\right)ds.$$

The likelihood functions are derived in the following theorem.

**Theorem 4** *Suppose that the log price Y_t_ and the volatility* $\sigma_{t}^{2}$ *satisfy* (5) *and* (4), *respectively*. *Let* $O\overset{d}{=}O_{i}=Y_{i-1}$, $C\overset{d}{=}C_{i}=Y_{i}$, $U\overset{d}{=}U_{i}=\max_{i-1\leq t\leq i}Y_{t}$ *and* $L\overset{d}{=}L_{i}=\min_{i-1\leq t\leq i}Y_{t}$. *Then, the joint densities of* (*U*, *C*), (*L*, *C*), *and* (*U*, *L*, *C*) *conditional on O* = *o are respectively*

$$\begin{array}{ccc} f_{U,C|O}\left(u,c|o\right) & = & \frac{2(2u-o-c)}{\sqrt{2\pi }}\frac{\Gamma \left(\alpha +3/2\right)\beta^{\alpha }2^{\alpha +3/2}}{\Gamma \left(\alpha \right){\left[{\left(2u-c-o\right)}^{2}+\mu^{2}-2\mu \left(c-o\right)+2\beta \right]}^{\alpha +3/2}},\\ f_{L,C|O}\left(\ell ,c|o\right) & = & \frac{2(o+c-2\ell )}{\sqrt{2\pi }}\frac{\Gamma \left(\alpha +3/2\right)\beta^{\alpha }2^{\alpha +3/2}}{\Gamma \left(\alpha \right){\left[{\left(o+c-2\ell \right)}^{2}+\mu^{2}-2\mu \left(c-o\right)+2\beta \right]}^{\alpha +3/2}},\\ f_{U,L,C|O}\left(u,\ell ,c|o\right) & = & \sum_{k=-\infty }^{\infty }\frac{4k\left(k+1\right)\beta^{\alpha }}{\sqrt{2\pi }\Gamma \left(\alpha \right)}g_{1}-\sum_{k=-\infty }^{\infty }\frac{4k^{2}\beta^{\alpha }}{\sqrt{2\pi }\Gamma \left(\alpha \right)}g_{2} \end{array}$$

*where ℓ* ≤ *c*, *o* ≤ *u and*

$$\begin{array}{ccc} g_{1} & = & \frac{\Gamma \left(\alpha +3/2\right)2^{\alpha +3/2}}{{\left[{\left(c+o-2u-2k\left(u-\ell \right)\right)}^{2}+\mu^{2}-2\mu \left(c-o\right)+2\beta \right]}^{\alpha +3/2}}\\ & & -\frac{{\left(c+o-2u-2k\left(u-\ell \right)\right)}^{2}\Gamma \left(\alpha +5/2\right)2^{\alpha +5/2}}{{\left[{\left(c+o-2u-2k\left(u-\ell \right)\right)}^{2}+\mu^{2}-2\mu \left(c-o\right)+2\beta \right]}^{\alpha +5/2}},\\ g_{2} & = & \frac{\Gamma \left(\alpha +3/2\right)2^{\alpha +3/2}}{{\left[{\left(c-o-2k\left(u-\ell \right)\right)}^{2}+\mu^{2}-2\mu \left(c-o\right)+2\beta \right]}^{\alpha +3/2}}\\ & & -\frac{{\left(c-o-2k\left(u-\ell \right)\right)}^{2}\Gamma \left(\alpha +5/2\right)2^{\alpha +5/2}}{{\left[{\left(c-o-2k\left(u-\ell \right)\right)}^{2}+\mu^{2}-2\mu \left(c-o\right)+2\beta \right]}^{\alpha +5/2}}. \end{array}$$

## Simulations

We construct the observations as follows. Set the *i*-th intra-daily log price to satisfy

$$\begin{array}{c} Y_{t_{i}+\Delta }=Y_{t_{i}}+\mu \Delta +\sigma W_{\Delta },\;0\leq t_{i}\leq 1, \end{array}$$

where *W*_Δ_ is normally distributed with mean 0 and variance Δ, and the sampling frequency is Δ = 1/5000. The log opening, maximum, minimum, and closing prices are denoted by *Y*_*i*_ = (*O*_*i*_, *U*_*i*_, *L*_*i*_, *C*_*i*_), where 1 ≤ *i* ≤ *n* with *n* = 250, say. Set *C*_*i*_ = *O*_*i*+1_, and repeat the above procedure for *i* = 1, 2, …, *n* − 1. We consider three practically oriented experiments based on the real observable data. According to the empirical evidence, the higher annualized market volatility is around 0.24, in contrast, the lower one is around 0.04. We also consider one particular case of the moderate volatility with the annualized market volatility being 0.12, and two cases of more violent volatilities with the annualized market volatilities being 0.36 and 0.48. So the daily volatilities are given by 0.04/250, 0.12/250, 0.24/250, 0.36/250, and 0.48/250. In addition, for the setting of drift term, we study two cases for the coefficient of variation: *σ*/*μ* = 1 (unit dispersion) and *σ*/*μ* = 2 (over dispersion).

We propose the MLE $\hat{\mu }$ for *μ* and $\left({\hat{\sigma }}_{u}^{2},{\hat{\sigma }}_{\ell }^{2}\right)$ for *σ*^2^ in Proposition 1. Theorem 3 provides the MLE ${\hat{\mu }}_{all}$ and ${\hat{\sigma }}_{all}^{2}$ for *μ* and *σ*^2^, respectively. For comparison, we consider the conventional MLE for *σ*^2^ based on discrete time closing prices given by

$$\begin{array}{c} s^{2}=\frac{1}{n-1}\sum_{i=2}^{n}{\left(R_{i}-\bar{R}\right)}^{2}, \end{array}$$

where *R*_*i*_ = *C*_*i*_ − *C*_*i*−1_ are the log returns of closing prices and $\bar{R}={\left(n-1\right)}^{-1}\sum_{i=2}^{n}R_{i}$. After 1000 replications, the relative error (RE, see for instance Helfrick and Cooper [21]) can be defined as

$$\begin{array}{c} \text{RE}=\frac{\text{RMSE}}{\text{True}\;\text{value}}, \end{array}$$

where RMSE stands for the root mean square error between the estimators and the true values. The values of $\hat{\mu }$, ${\hat{\mu }}_{all}$, ${\hat{\sigma }}_{u}^{2}$, ${\hat{\sigma }}_{\ell }^{2}$, ${\hat{\sigma }}_{all}^{2}$, and *s*^2^ are shown in Tables 1 and 2. We can see that the RE of $\hat{\mu }$ is slightly less than that of ${\hat{\mu }}_{all}$. This is possibly caused by truncating the infinite series into a finite sum of ±20 terms. On the other hand, the REs of ${\hat{\sigma }}_{u}^{2},{\hat{\sigma }}_{\ell }^{2}$, and ${\hat{\sigma }}_{all}^{2}$ are much less than *s*^2^. In particular, the performance of ${\hat{\sigma }}_{all}^{2}$ is the best in terms of the smallest RE. Meanwhile, the relative efficiencies of *s*^2^ compared to ${\hat{\sigma }}_{u}^{2}$ and ${\hat{\sigma }}_{\ell }^{2}$ are written as $\frac{{\text{RE}}_{s^{2}}}{{\text{RE}}_{{\hat{\sigma }}_{u}^{2}}}$ and $\frac{{\text{RE}}_{s^{2}}}{{\text{RE}}_{{\hat{\sigma }}_{\ell }^{2}}}$ ranging from 1.55 to 1.69. Furthermore, the relative efficiencies of *s*^2^ compared to ${\hat{\sigma }}_{all}^{2}$ given by $\frac{{\text{RE}}_{s^{2}}}{{\text{RE}}_{{\hat{\sigma }}_{all}^{2}}}$ are ranging from 1.74 to 2.11. The results indicate that the proposed estimators (${\hat{\sigma }}_{u}^{2}$, ${\hat{\sigma }}_{\ell }^{2}$, and ${\hat{\sigma }}_{all}^{2}$) display substantial improvements and is stable in different scenarios. We conclude that the easy to implement estimator $\hat{\mu }$ has lower relative error than ${\hat{\mu }}_{all}$. In addition, the whole observations-based estimator ${\hat{\sigma }}_{all}^{2}$ has better accuracy than ${\hat{\sigma }}_{u}^{2}$, ${\hat{\sigma }}_{\ell }^{2}$, and even the conventional *s*^2^, in terms of relative error.

**Table 1:** REs (in percentages) of $\hat{\mu }$, ${\hat{\mu }}_{all}$, ${\hat{\sigma }}_{u}^{2}$, ${\hat{\sigma }}_{\ell }^{2}$, ${\hat{\sigma }}_{all}^{2}$, and *s*^2^, then the relative efficiencies between *s*^2^ and the alternatives.

	*σ*/*μ* = 1				
	*σ*^2^ = 0.04/250	*σ*^2^ = 0.12/250	*σ*^2^ = 0.24/250	*σ*^2^ = 0.36/250	*σ*^2^ = 0.48/250
RE of $\hat{\mu }$	6.51	6.07	6.31	6.31	6.33
RE of ${\hat{\mu }}_{all}$	7.29	6.53	6.84	6.31	6.33
RE of ${\hat{\sigma }}_{u}^{2}$	5.71	5.57	5.48	5.51	5.66
RE of ${\hat{\sigma }}_{\ell }^{2}$	5.40	5.41	5.66	5.49	5.74
RE of ${\hat{\sigma }}_{all}^{2}$	4.99	5.01	5.29	4.31	4.57
RE of *s*^2^	8.83	8.73	9.19	8.69	8.85
Eff of $s^{2}/{\hat{\sigma }}_{u}^{2}$	1.55	1.57	1.68	1.58	1.56
Eff of $s^{2}/{\hat{\sigma }}_{\ell }^{2}$	1.63	1.61	1.62	1.58	1.54
Eff of $s^{2}/{\hat{\sigma }}_{all}^{2}$	1.77	1.74	1.74	2.02	1.94

**Table 2:** REs (in percentages) of $\hat{\mu }$, ${\hat{\mu }}_{all}$, ${\hat{\sigma }}_{u}^{2}$, ${\hat{\sigma }}_{\ell }^{2}$, ${\hat{\sigma }}_{all}^{2}$, and *s*^2^, then the relative efficiencies between *s*^2^ and the alternatives.

	*σ*/*μ* = 2				
	*σ*^2^ = 0.04/250	*σ*^2^ = 0.12/250	*σ*^2^ = 0.24/250	*σ*^2^ = 0.36/250	*σ*^2^ = 0.48/250
RE of $\hat{\mu }$	12.69	12.39	12.57	12.53	12.68
RE of ${\hat{\mu }}_{all}$	12.72	12.39	12.76	12.74	13.35
RE of ${\hat{\sigma }}_{u}^{2}$	5.39	5.56	5.32	5.46	5.48
RE of ${\hat{\sigma }}_{\ell }^{2}$	5.55	5.51	5.33	5.32	5.70
RE of ${\hat{\sigma }}_{all}^{2}$	4.56	4.31	4.99	5.16	4.39
RE of *s*^2^	9.03	9.11	9.02	8.69	9.06
Eff of $s^{2}/{\hat{\sigma }}_{u}^{2}$	1.67	1.64	1.69	1.66	1.65
Eff of $s^{2}/{\hat{\sigma }}_{\ell }^{2}$	1.63	1.65	1.69	1.70	1.59
Eff of $s^{2}/{\hat{\sigma }}_{all}^{2}$	1.98	2.11	1.81	1.76	2.06

For the stochastic volatility model, the parameters for the volatility term (cf. (4)) are given by *μ* = 0, *ρ* = 1, *α* = 5, and *β* = 4*V* with *V* = 0.04/250 (low volatility case), 0.12/250 (moderate volatility case), and 0.24/250 (high volatility case). Note that the term $V=\frac{\beta }{\alpha -1}$ represents the long term means of the volatility processes.

We obtain the MLEs $\hat{\alpha }$ and $\hat{\beta }$ via Theorem 4 and the MLE for ${\hat{\sigma }}_{v}$ given by ${\hat{\sigma }}_{v}=\hat{\beta }/\left(\hat{\alpha }-1\right)$. We then consider the conventional estimator *s*^2^, ${\hat{\sigma }}_{all}^{2}$ discussed in Theorem 3 for the constant volatility case, and the volatility estimator proposed by Chou [10, 11], denoted as ${\hat{\sigma }}_{C}^{2}$ for comparison. Since the volatility estimator proposed by Chou [10, 11] based on the ranges, upward ranges, and downward ranges are quite similar, we only discuss one particular case among them. For simplify, we intend to fit GARCH(1,1) for ${\hat{\sigma }}_{C}^{2}$ and the results are shown in Table 3. In the case of the high volatility, the relative efficiencies of *s*^2^ compared to ${\hat{\sigma }}_{v}$, ${\hat{\sigma }}_{all}^{2}$, and ${\hat{\sigma }}_{C}^{2}$ are 2.19, 1.60, and 1.81, respectively.

**Table 3:** REs (in percentages) of ${\hat{\sigma }}_{v}^{2}$, ${\hat{\sigma }}_{all}^{2}$, ${\hat{\sigma }}_{C}^{2}$, and *s*^2^, then the relative efficiencies between *s*^2^ and the alternatives.

	*V* = 0.04/250	*V* = 0.12/250	*V* = 0.24/250	*V* = 0.36/250	*V* = 0.48/250
RE of ${\hat{\sigma }}_{v}^{2}$	4.56	4.73	4.66	4.82	4.57
RE of ${\hat{\sigma }}_{all}^{2}$	6.31	6.32	6.37	6.42	6.31
RE of ${\hat{\sigma }}_{C}^{2}$	40.95	40.39	5.65	30.95	32.23
RE of *s*^2^	10.21	10.66	10.22	10.57	10.18
Eff of $s^{2}/{\hat{\sigma }}_{v}^{2}$	2.24	2.25	2.19	2.19	2.23
Eff of $s^{2}/{\hat{\sigma }}_{all}^{2}$	1.62	1.69	1.60	1.65	1.61
Eff of $s^{2}/{\hat{\sigma }}_{C}^{2}$	0.25	0.26	1.81	0.34	0.32

As we expected that more information (maximum/minimum prices) improve the accuracy of the estimation of the volatility. Besides, the estimators ${\hat{\sigma }}_{v}$ and ${\hat{\sigma }}_{C}^{2}$ estimated under the stochastic volatility model perform better than the one based on the constant volatility model written as ${\hat{\sigma }}_{all}^{2}$ estimated in the constant volatility case. Meanwhile, ${\hat{\sigma }}_{v}$ has the lowest relative errors since it is obtained from the exact likelihood function instead of the quasi-likelihood function. For the moderate and low volatility cases, the estimators ${\hat{\sigma }}_{v}$ are still the best one with the lowest relative errors. Note that the estimator ${\hat{\sigma }}_{C}^{2}$ performs worse in the case of the moderate and low volatility cases. It may be due to the fewer observations or inadequate lags for the GARCH model. This is beyond the scope of this study and we omit the further discussion on it.

## Real application

We present the one-step predictions of an interval valued time series for the S&P 500 index. According to Arroyo et al. [4], the daily high/low prices of the S&P 500 index are utilized to compare the prediction performances of various methods. We make an one-step prediction by applying the rolling window where the historical data of previous year is used to estimate the parameters. The most challenging period is the financial crisis occurred on year 2008. Therefore, we first study the performances of various methods in one-step prediction on year 2008. Besides, we want to investigate the effect on the historical data. We select the periods of 2006 and 2017. For the former, the historical data of previous year (2005) has the similar pattern as the current year (2006). For the latter, the volatility in the historical data (2016) is violent compared to the predicted period (2017). Therefore, the prediction and estimation time periods are set to be

1. similar volatility period: prediction from January 2006 to December 2006; estimation from January 2005 to December 2005.
2. High volatility period: prediction from January 2008 to December 2008; estimation from January 2007 to December 2007.
3. dissimilar volatility period: prediction from January 2017 to December 2017; estimation from January 2016 to December 2016.

**Fig 1:**
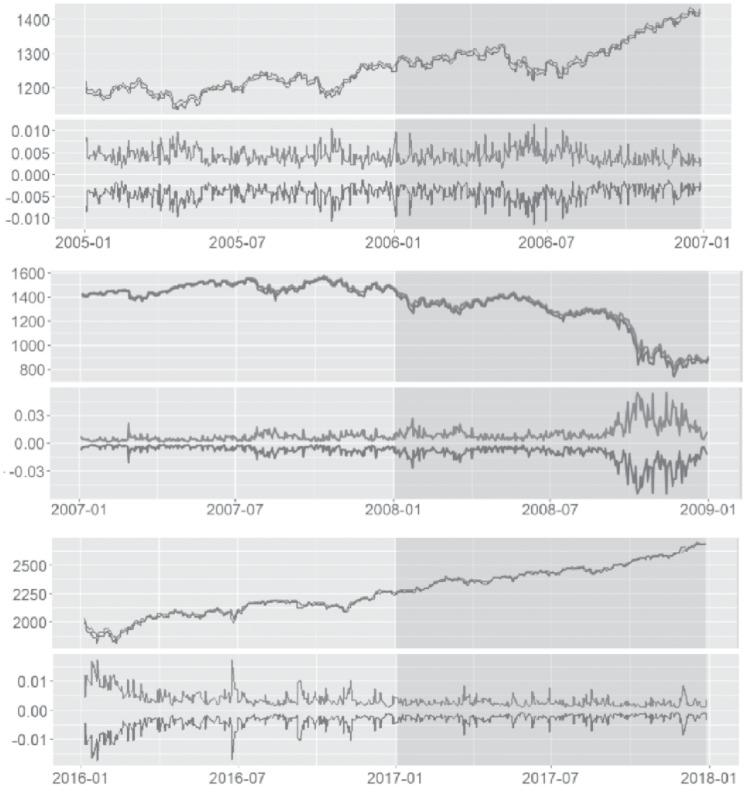
The maximum and minimum prices with the corresponding centralized maximum and minimum returns for the similar volatility period (upper panel), high volatility period (middle panel), and dissimilar volatility period (lower panel).

Fig 1 depicts the maximum/minimum prices with the corresponding centralized maximum/minimum returns (denoted by the difference between the logarithmic maximum/minimum and opening prices) in these three periods. From Fig 1, we can see that the volatility in the beginning of 2016 is higher than the whole year of 2017. Meanwhile, the volatilities have no significant difference between the years of 2005 and 2006. In the end of 2008, of course, the volatility is much violent than the usual situation.

In order to quantify the accuracy of the one-step forecast, we adopt the measure of the mean distance error (MDE) defined as

$$\begin{array}{c} MDE=(\sum_{t=1}^{T}\frac{{\left(U_{t}-{\hat{U}}_{t}\right)}^{2}+{\left(L_{t}-{\hat{L}}_{t}\right)}^{2}}{2T}{)}^{1/2}, \end{array}$$

where *X*_*t*_ = [*L*_*t*_, *U*_*t*_] is the true interval valued data and ${\hat{X}}_{t}=\left[{\hat{L}}_{t},{\hat{U}}_{t}\right]$ is the estimated one. Following Rodrigues and Salish (2011), descriptive statistics are also evaluated by

1. coverage rate: $R_{C}=T^{-1}\sum_{t=1}^{T}\frac{w(X_{t}\cap {\hat{X}}_{t})}{w(X_{t})}$,
2. efficient rate: $R_{E}=T^{-1}\sum_{t=1}^{T}\frac{w(X_{t}\cap {\hat{X}}_{t})}{w({\hat{X}}_{t})}$,
3. normalized symmetric difference: $R_{N}=T^{-1}\sum_{t=1}^{T}\frac{w(X_{t}\cap {\hat{X}}_{t})}{w(X_{t}\cup {\hat{X}}_{t})}$,

where *w*(*X*) represents the length of an interval *X*. By using Proposition 2, we obtain the high and low prices by one-step forecasting. Then we can compare our results with those of Naïve method, EWMA, k-NN, VAR(3), VECM(3), (cf. Arroyo et al. [4]) and CR-SETAR (cf. Rodrigues and Salish [5]). Let [*U*_1_, *L*_1_],…,[*U*_*n*_, *L*_*n*_] be the observations and our goal is to forecast the interval on day *n* + 1, i.e., $\left[{\hat{U}}_{n+1},{\hat{L}}_{n+1}\right]$. The Naïve method predicts the intervals by using the previous one, that is, $\left[{\hat{U}}_{n+1},{\hat{L}}_{n+1}\right]=\left[U_{n},L_{n}\right]$. EWMA provides the predicted interval as follows

$$\begin{array}{c} \left[{\hat{U}}_{n+1},{\hat{L}}_{n+1}\right]=\sum_{j=0}^{n-1}\lambda {\left(1-\lambda \right)}^{j}\left[U_{n-j},L_{n-j}\right]. \end{array}$$

We set λ = 0.04 as suggested by Arroyo et al. [4]. The k-NN method is to find the historical *k* sequences with *d* points which are closest ones to the current ones in terms of MDEs, and then is to evaluate the average of the consecutive intervals of these *k* closest sequences. Let ${\left[U,L\right]}_{t}^{\left(d\right)}={\left(\left[U_{t},L_{t}\right],\ldots ,\left[U_{t-(d-1)},L_{t-(d-1)}\right]\right)}^{\prime }$ be the *d*-dimensional interval-valued vectors. Then, we locate ${\left[U,L\right]}_{t_{1}}^{\left(d\right)},\ldots ,{\left[U,L\right]}_{t_{k}}^{\left(d\right)}$ for *t*_1_ < ⋯ < *t*_*k*_ < *n* in order to have the smallest MDEs compared to ${\left[U,L\right]}_{n}^{\left(d\right)}$. Then, the predicted interval based on the equal weights k-NN method, denoted as k-NN(eq), is given by

$$\begin{array}{c} \left[{\hat{U}}_{n+1},{\hat{L}}_{n+1}\right]=\frac{1}{k}\sum_{j=1}^{k}\left[U_{t_{j}+1},L_{t_{j}+1}\right]. \end{array}$$

Further, the proportion weights k-NN method, denoted as k-NN(prop), is given by

$$\begin{array}{c} \left[{\hat{U}}_{n+1},{\hat{L}}_{n+1}\right]=\sum_{j=1}^{k}w_{j}\left[U_{t_{j}+1},L_{t_{j}+1}\right], \end{array}$$

where $w_{j}=\psi_{j}/\left(\sum_{m=1}^{k}\psi_{m}\right)$. *ψ*_*j*_ is defined as the inverse of the MDE between ${\left[U,L\right]}_{n}^{\left(d\right)}$ and ${\left[U,L\right]}_{t_{j}}^{\left(d\right)}$ plus a small constant, say 10^−8^. Referred to Arroyo et al. [4], *d* = 2 for three consideration periods and *k* = 23, 18, and 26 for the similar volatility, high volatility, and dissimilar volatility periods, respectively. According to the results in Arroyo et al. [4], the VAR(3) model based on the vector of differenced center and radius time series can be written as

$$\begin{array}{c} (\begin{array}{c} \Delta C_{t}\\ R_{t} \end{array})=(\begin{array}{c} \beta_{c}\\ \beta_{r} \end{array})+\sum_{j=1}^{3}(\begin{array}{cc} \beta_{c,j}^{c} & \beta_{r,j}^{c}\\ \beta_{c,j}^{r} & \beta_{r,j}^{r} \end{array})(\begin{array}{c} \Delta C_{t-j}\\ R_{t-j} \end{array})+(\begin{array}{c} \epsilon_{c,t}\\ \epsilon_{r,t} \end{array}), \end{array}$$

where *C*_*t*_ = (*U*_*t*_ + *L*_*t*_)/2, *R*_*t*_ = (*U*_*t*_ − *L*_*t*_)/2, and Δ*C*_*t*_ = *C*_*t*_ − *C*_*t*−1_. Using the historical observations to fit the VAR(3) model and obtain all of the parameter estimations, the predicted interval is $\left[{\hat{U}}_{n+1},{\hat{L}}_{n+1}\right]=\left[{\hat{C}}_{n+1}+{\hat{R}}_{n+1},{\hat{C}}_{n+1}-{\hat{R}}_{n+1}\right]$, where ${\hat{C}}_{n+1}=\Delta {\hat{C}}_{n+1}+C_{n}$ and $\left(\Delta {\hat{C}}_{n+1},\;{\hat{R}}_{n+1}\right)$ satisfies

$$\begin{array}{c} (\begin{array}{c} \Delta {\hat{C}}_{n+1}\\ {\hat{R}}_{n+1} \end{array})=(\begin{array}{c} {\hat{\beta }}_{c}\\ {\hat{\beta }}_{r} \end{array})+\sum_{j=1}^{3}(\begin{array}{cc} {\hat{\beta }}_{c,j}^{c} & {\hat{\beta }}_{r,j}^{c}\\ {\hat{\beta }}_{c,j}^{r} & {\hat{\beta }}_{r,j}^{r} \end{array})(\begin{array}{c} \Delta C_{n+1-j}\\ R_{n+1-j} \end{array}). \end{array}$$

Next, assuming that (*U*_*t*_, *L*_*t*_) satisfies the VECM(3) model, this implies

$$\begin{array}{c} (\begin{array}{c} \Delta U_{t}\\ \Delta L_{t} \end{array})=(\begin{array}{c} \beta_{u}\\ \beta_{l} \end{array})+\Pi (\begin{array}{c} U_{t-1}\\ L_{t-1} \end{array})+\sum_{j=1}^{3}(\begin{array}{cc} \beta_{u,j}^{u} & \beta_{l,j}^{u}\\ \beta_{u,j}^{l} & \beta_{l,j}^{l} \end{array})(\begin{array}{c} \Delta U_{t-j}\\ \Delta L_{t-j} \end{array})+(\begin{array}{c} \epsilon_{u,t}\\ \epsilon_{l,t} \end{array}), \end{array}$$

where Δ*U*_*t*_ = *U*_*t*_ − *U*_*t*−1_ and Δ*L*_*t*_ = *L*_*t*_ − *L*_*t*−1_. Using the historical observations to fit the VECM(3) model and to obtain all of the parameter estimations, the predicted interval is $\left[{\hat{U}}_{n+1},{\hat{L}}_{n+1}\right]=\left[\Delta {\hat{U}}_{n+1}+U_{n},\Delta {\hat{L}}_{n+1}+L_{n}\right]$, where $\left(\Delta {\hat{U}}_{n+1},\Delta {\hat{L}}_{n+1}\right)$ satisfies

$$\begin{array}{c} (\begin{array}{c} \Delta {\hat{U}}_{n+1}\\ \Delta {\hat{L}}_{n+1} \end{array})=(\begin{array}{c} {\hat{\beta }}_{u}\\ {\hat{\beta }}_{l} \end{array})+\hat{\Pi }(\begin{array}{c} U_{n}\\ L_{n} \end{array})+\sum_{j=1}^{3}(\begin{array}{cc} {\hat{\beta }}_{u,j}^{u} & {\hat{\beta }}_{l,j}^{u}\\ {\hat{\beta }}_{u,j}^{l} & {\hat{\beta }}_{l,j}^{l} \end{array})(\begin{array}{c} \Delta U_{t-j}\\ \Delta L_{t-j} \end{array}). \end{array}$$

Following Rodrigues and Salish [5], the two-regime CR-SETAR model based on center and radius time series is

$$\begin{array}{ccc} \left(\begin{array}{c} C_{t}\\ R_{t} \end{array}\right) & = & [(\begin{array}{c} \alpha_{c}\\ \alpha_{r} \end{array})+\sum_{j=1}^{p}(\begin{array}{cc} \alpha_{c,j}^{c} & \alpha_{r,j}^{c}\\ \alpha_{c,j}^{r} & \alpha_{r,j}^{r} \end{array})(\begin{array}{c} C_{t-j}\\ R_{t-j} \end{array})]I_{\left\{R_{t-d}\leq \gamma \right\}}\\ & & +[(\begin{array}{c} \beta_{c}\\ \beta_{r} \end{array})+\sum_{j=1}^{q}(\begin{array}{cc} \beta_{c,j}^{c} & \beta_{r,j}^{c}\\ \beta_{c,j}^{r} & \beta_{r,j}^{r} \end{array})(\begin{array}{c} C_{t-j}\\ R_{t-j} \end{array})]I_{\left\{R_{t-d}>\gamma \right\}}+(\begin{array}{c} \epsilon_{c,t}\\ \epsilon_{r,t} \end{array}), \end{array}$$

where *I*_{}_ represents the indicator function. We choose *p* = 6, *q* = 8, and *d* = 1 same as the cases proposed by Rodrigues and Salish [5]. Using the historical observations to fit the CR-SETAR model and obtain all of the parameter estimations, the predicted interval is $\left[{\hat{U}}_{n+1},{\hat{L}}_{n+1}\right]=\left[{\hat{C}}_{n+1}+{\hat{R}}_{n+1},{\hat{C}}_{n+1}-{\hat{R}}_{n+1}\right]$, where $\left({\hat{C}}_{n+1},{\hat{R}}_{n+1}\right)$ satisfies

$$\begin{array}{ccc} \left(\begin{array}{c} {\hat{C}}_{n+1}\\ {\hat{R}}_{n+1} \end{array}\right) & = & [(\begin{array}{c} {\hat{\alpha }}_{c}\\ {\hat{\alpha }}_{r} \end{array})+\sum_{j=1}^{6}(\begin{array}{cc} {\hat{\alpha }}_{c,j}^{c} & {\hat{\alpha }}_{r,j}^{c}\\ {\hat{\alpha }}_{c,j}^{r} & {\hat{\alpha }}_{r,j}^{r} \end{array})(\begin{array}{c} C_{t-j}\\ R_{t-j} \end{array})]I_{\left\{R_{n-1}\leq \hat{\gamma }\right\}}\\ & & +[(\begin{array}{c} {\hat{\beta }}_{c}\\ {\hat{\beta }}_{r} \end{array})+\sum_{j=1}^{8}(\begin{array}{cc} {\hat{\beta }}_{c,j}^{c} & {\hat{\beta }}_{r,j}^{c}\\ {\hat{\beta }}_{c,j}^{r} & {\hat{\beta }}_{r,j}^{r} \end{array})(\begin{array}{c} C_{t-j}\\ R_{t-j} \end{array})]I_{\left\{R_{n-1}>\hat{\gamma }\right\}}. \end{array}$$

Based on the results of Tables 4, 5 and 6 in terms of the MDE measurement, our proposed method gives the better prediction based on the smaller MDE. Compared to the Naïve method, the improvement for MDE is around 26%, 23%, and 38% on the similar volatility period, high volatility period, and dissimilar volatility period, respectively. Meanwhile, compared to the best one among other methods, they are 17%, 20%, and 35% on the similar volatility period, high volatility period, and dissimilar volatility period, respectively. In addition, the measurements *R*_*E*_ and *R*_*N*_ of our proposed prediction method are also the largest one. The above results show that the proposed model presents the more accurate interval financial time series in the real world.

**Table 4:** Comparison between our proposed method and alternatives during the similar volatility period.

	MDE	*R*_*C*_	*R*_*E*_	*R*_*N*_
Naïve	7.056	58.93	59.88	42.43
EWMA	6.741	66.50	54.60	43.30
k-NN(eq)	6.429	66.51	54.86	44.29
k-NN(prop)	6.303	65.93	55.99	44.82
VAR	6.611	67.21	55.86	43.99
VECM	6.547	66.42	55.40	43.72
CR-SETAR	9.052	**91.22**	43.46	41.08
Proposed method	**5.232**	74.42	**62.67**	**50.34**

**Table 5:** Comparison between our proposed method and alternatives during the high volatility period.

	MDE	*R*_*C*_	*R*_*E*_	*R*_*N*_
Naïve	21.643	56.95	56.12	38.68
EWMA	21.174	65.32	59.33	40.85
k-NN(eq)	21.146	46.82	57.07	37.05
k-NN(prop)	21.734	45.86	56.21	36.52
VAR	20.788	57.98	60.64	38.67
VECM	20.833	61.27	60.39	39.40
CR-SETAR	27.520	**86.47**	42.96	40.01
Proposed method	**16.723**	63.69	**69.17**	**47.56**

**Table 6:** Comparison between our proposed method and alternatives during the dissimilar volatility period.

	MDE	*R*_*C*_	*R*_*E*_	*R*_*N*_
Naïve	9.671	52.54	49.71	36.22
EWMA	9.314	56.89	47.01	37.62
k-NN(eq)	9.560	60.52	46.21	38.09
k-NN(prop)	9.550	60.51	46.30	38.37
VAR	9.583	59.06	47.10	38.29
VECM	9.424	52.58	49.16	36.86
CR-SETAR	9.779	60.96	46.33	38.63
Proposed method	**6.039**	**82.29**	**59.82**	**51.20**

## Conclusion

We propose the joint densities of daily log opening, maximum and closing prices and daily log opening, minimum and closing prices based on stochastic differential equations. Simulation studies show that the proposed estimators have higher efficiency than the conventional one using RE. In the real data analysis for S&P 500 index, the one-step forecasts of proposed method outperforms than several alternatives in terms of MDE, *R*_*E*_, and *R*_*N*_.

The proposed methodology has several interesting extensions. In this paper, we study the stochastic volatility model on discrete time where the stochastic volatility is driven by a stationary distribution during a fixed time interval. In the literature, it is nature to consider the intra-daily volatility is governed by stochastic processes. However, owing to the stochasticity feature of the volatility, the Girsanov theorem can not be applied straightforwardly. Based on Akahori et al. [22], during the small time interval, the asymptotic results can be used to simplify the Girsanov theorem by using the Taylor expansion. Then the likelihood function can be derived and the corresponding maximum likelihood estimators can be obtained. We left this issue as our future project. Alternatively, from the investment strategy point of view, it is also interesting to study the high dimensional financial interval time series for multiple assets leading to the corresponding estimation problem for the proposed high dimensional model.

## Appendix: Proofs

### Proof of Theorem 1

Let *Y*_*t*_ = log *S*_*t*_ given by the dynamics

$$\begin{array}{c} dY_{t}=\mu dt+\sigma dW_{t},\;Y_{0}=o. \end{array}$$ (A.1)

Let *M*_*t*_ = sup_0≤*s*≤*t*_ *Y*_*s*_. The joint cdf of *Y*_*t*_ and *M*_*t*_ is written as

$$\begin{array}{ccc} & & P(M_{t}<m,Y_{t}<y|Y_{0}=o)\\ & = & E[I_{\left\{M_{t}<m,Y_{t}<y\right\}}|Y_{0}=o]\\ & = & E[I_{\left\{\frac{M_{t}-o}{\sigma }<\frac{m-o}{\sigma },\frac{Y_{t}-o}{\sigma }<\frac{y-o}{\sigma }\right\}}|Y_{0}=o]. \end{array}$$

Let ${\tilde{Y}}_{t}=\frac{Y_{t}-o}{\sigma }$ given by

$$\begin{array}{c} d{\tilde{Y}}_{t}=\frac{\mu }{\sigma }dt+dW_{t},\;{\tilde{Y}}_{0}=0 \end{array}$$

and ${\tilde{M}}_{t}=\sup_{0\leq s\leq t}{\tilde{Y}}_{s}$. Applying the Girsanov theorem implies

$$\begin{array}{ccc} & & E[I_{\left\{\frac{M_{t}-o}{\sigma }<\frac{m-o}{\sigma },\frac{Y_{t}-o}{\sigma }<\frac{y-o}{\sigma }\right\}}|Y_{0}=o]\\ & = & E[I_{\left\{{\tilde{M}}_{t}<\frac{m-o}{\sigma },{\tilde{Y}}_{t}<\frac{y-o}{\sigma }\right\}}|Y_{0}=o]\\ & = & E[I_{\left\{{\tilde{M}}_{t}<\frac{m-o}{\sigma },W_{t}<\frac{y-o}{\sigma }\right\}}e^{\frac{\mu }{\sigma }W_{t}-\frac{\mu^{2}}{2\sigma^{2}}t}|Y_{0}=o] \end{array}$$

where *W*_*t*_ is a standard Brownian motion. Hence, using the joint pdf of *W*_*t*_ and sup_0≤*s*≤*t*_ *W*_*s*_, we obtain the joint density of *Y*_*t*_ and *M*_*t*_ is written as

$$\begin{array}{ccc} & & P(M_{t}<m,Y_{t}<y)\\ & = & e^{-\frac{\mu^{2}}{2\sigma^{2}}t}\int_{0}^{\frac{m-o}{\sigma }}\int_{-\infty }^{\frac{y-o}{\sigma }}e^{\frac{\mu }{\sigma }w}\frac{2(2z-w)}{t\sqrt{2\pi t}}e^{-\frac{{\left(2z-w\right)}^{2}}{2t}}dwdz,\;y\leq m,\;o\leq m. \end{array}$$

The corresponding joint density function is given by

$$\begin{array}{c} f_{M_{t},Y_{t}}\left(m,y\right)=\frac{2(2m-y-o)}{t\sigma^{3}\sqrt{2\pi t}}\;\text{exp}\;\{-\frac{\mu^{2}}{2\sigma^{2}}t+\frac{\mu (y-o)}{\sigma^{2}}-\frac{{\left(2m-y-o\right)}^{2}}{2\sigma^{2}t}\},y\leq m,\;o\leq m. \end{array}$$

Using *t* = *i* − (*i* − 1) = 1, we obtain (2).

### Proof of Theorem 2

Define the minimum to date for Brownian motion to be

$$\begin{array}{c} m_{t}=\min_{0\leq s\leq t}W_{s}. \end{array}$$

By reflection principle, we have

$$\begin{array}{c} P\left(m_{t}\leq l,\;W_{t}\geq w\right)=P\left(W_{t}\leq 2l-w\right). \end{array}$$ (A.2)

Eq (A.2) implies

$$\begin{array}{c} \int_{-\infty }^{l}\int_{w}^{\infty }f_{m,W}\left(x,y\right)dydx=\frac{1}{\sqrt{2\pi t}}\int_{-\infty }^{2l-w}e^{-\frac{z^{2}}{2t}}dz. \end{array}$$ (A.3)

By differentiating the Eq (A.3) with respect to *l* and *w*, we obtain

$$\begin{array}{c} f_{m,W}\left(l,w\right)=\frac{2(w-2l)}{t\sqrt{2\pi t}}\;\text{exp}\;\{-\frac{{\left(w-2l\right)}^{2}}{2t}\},\;w\geq l,\;l<0. \end{array}$$

The rest part follows the same procedure as Theorem 1 to demonstrate this proof.

### Proof of Theorem 3

Given

$$\begin{array}{c} dY_{t}=\mu dt+\sigma dW_{t},\;Y_{0}=o, \end{array}$$

$M_{t}^{u}=\sup_{0\leq s\leq t}Y_{s}$ and $M_{t}^{l}=\inf_{0\leq s\leq t}Y_{s}$, Similarly, applying the Girsanov theorem and using ${\tilde{Y}}_{t}=\frac{Y_{t}-o}{\sigma }$ given by

$$\begin{array}{c} d{\tilde{Y}}_{t}=\frac{\mu }{\sigma }dt+dW_{t},\;{\tilde{Y}}_{0}=0, \end{array}$$

and ${\tilde{M}}_{t}^{u}=\sup_{0\leq s\leq t}{\tilde{Y}}_{t}$ and ${\tilde{M}}_{t}^{l}=\inf_{0\leq s\leq t}{\tilde{Y}}_{t}$, we have

$$\begin{array}{ccc} & & P_{Y_{t},M_{t}^{u},M_{t}^{l}}(a\leq M_{t}^{l}\leq M_{t}^{u}\leq b,Y_{t}\in dy|Y_{0}=o)\\ & = & E[I_{\left\{a\leq M_{t}^{l}\leq M_{t}^{u}\leq b,Y_{t}\in dy\right\}}|Y_{0}=o]\\ & = & E[I_{\left\{\frac{a-o}{\sigma }\leq \frac{M_{t}^{l}-o}{\sigma }\leq \frac{M_{t}^{u}-o}{\sigma }\leq \frac{b-o}{\sigma },\frac{Y_{t}-o}{\sigma }\in dy\right\}}|Y_{0}=o]\\ & = & E[I_{\left\{\frac{a-o}{\sigma }\leq {\tilde{M}}_{t}^{l}\leq {\tilde{M}}_{t}^{u}\leq \frac{b-o}{\sigma },{\tilde{Y}}_{t}\in dy\right\}}|Y_{0}=o]\\ & = & E[I_{\left\{\frac{a-o}{\sigma }\leq {\tilde{M}}_{t}^{l}\leq {\tilde{M}}_{t}^{u}\leq \frac{b-o}{\sigma },W_{t}\in dy\right\}}e^{\frac{\mu }{\sigma }W_{t}-\frac{\mu^{2}}{2\sigma^{2}}t}|Y_{0}=o] \end{array}$$ (A.4)

where *W*_*t*_ is a standard Brownian motion. Hence, using (3) proposed by Choi and Roh (2013) and differentiating (A.4) with respect to *a* and *b*, we obtain

$$\begin{array}{ccc} f_{Y_{t},M_{t}^{u},M_{t}^{l}}\left(y,m^{u},m^{l}\right) & = & \sum_{k=-\infty }^{\infty }\frac{4k(k+1)}{t\sigma^{3}\sqrt{2\pi t}}(1-\frac{{\left(y+o-2m^{u}-2k\left(m^{u}-m^{l}\right)\right)}^{2}}{\sigma^{2}t})f_{1}\\ & & -\sum_{k=-\infty }^{\infty }\frac{4k^{2}}{t\sigma^{3}\sqrt{2\pi t}}(1-\frac{{\left(y-o-2k\left(m^{u}-m^{l}\right)\right)}^{2}}{\sigma^{2}t})f_{2} \end{array}$$

where *ℓ* ≤ *c*, *o* ≤ *u* and

$$\begin{array}{ccc} f_{1} & = & \text{exp}\;\{-\frac{{\left(y+o-2m^{u}-2k\left(m^{u}-m^{l}\right)\right)}^{2}}{2\sigma^{2}t}-\frac{\mu^{2}}{2\sigma^{2}}t+\frac{\mu (y-o)}{\sigma^{2}}\}\\ f_{2} & = & \text{exp}\;\{-\frac{{\left(y-o-2k\left(m^{u}-m^{l}\right)\right)}^{2}}{2\sigma^{2}t}-\frac{\mu^{2}}{2\sigma^{2}}t+\frac{\mu (y-o)}{\sigma^{2}}\}, \end{array}$$

where *m*^*l*^ ≤ *y* ≤ *m*^*u*^.

### Proof of Proposition 1

Based on the joint density of Theorem 1, the likelihood function of (*μ*, *σ*) based on observations $\left(\vec{u},\vec{o},\vec{c}\right)$ is

$$\begin{array}{c} L\left(\mu ,\sigma^{2}|\vec{u},\vec{o},\vec{c}\right)=\prod_{i=1}^{n}\frac{2(2u_{i}-o_{i}-c_{i})}{\sqrt{2\pi }\sigma^{3}}\;\text{exp}\;\{-\frac{{\left(2u_{i}-o_{i}-c_{i}\right)}^{2}}{2\sigma^{2}}-\frac{\mu^{2}}{2\sigma^{2}}+\frac{\mu (c_{i}-o_{i})}{\sigma^{2}}\},\;\;\; \end{array}$$ (A.5)

where $\vec{u}=\left(u_{1},\cdots ,u_{n}\right)$, $\vec{o}=\left(o_{1},\cdots ,o_{n}\right)$, and $\vec{c}=\left(c_{1},\cdots ,c_{n}\right).$ Take log function to both sides of (A.5), we have

$$\begin{array}{ccc} \ell (\mu ,\sigma^{2}|\vec{u},\vec{o},\vec{c}) & = & \sum_{i=1}^{n}\log\frac{2(2u_{i}-o_{i}-c_{i})}{\sqrt{2\pi }}-\frac{3n}{2}\log\sigma^{2}\\ & & -\sum_{i=1}^{n}\frac{{\left(2u_{i}-o_{i}-c_{i}\right)}^{2}+\mu^{2}-2\mu \left(c_{i}-o_{i}\right)}{2\sigma^{2}}. \end{array}$$ (A.6)

Differentiating the Eq (A.6) with respect to *μ*, it implies

$$\begin{array}{c} \frac{\partial }{\partial \mu }\ell \left(\mu ,\sigma^{2}|\vec{u},\vec{o},\vec{c}\right)=-\sum_{i=1}^{n}\left(\mu -\left(c_{i}-o_{i}\right)\right)/\sigma^{2}=0. \end{array}$$

Then, the maximum likelihood estimator of *μ* is

$$\begin{array}{c} \hat{\mu }=n^{-1}\sum_{i=1}^{n}\left(c_{i}-o_{i}\right). \end{array}$$ (A.7)

Next, Differentiating the Eq (A.6) with respect to *σ*^2^, we obtain

$$\begin{array}{c} \frac{\partial }{\partial \sigma^{2}}\ell \left(\mu ,\sigma^{2}|\vec{u},\vec{o},\vec{c}\right)=-\frac{3n}{2\sigma^{2}}+\sum_{i=1}^{n}\frac{{\left(2u_{i}-o_{i}-c_{i}\right)}^{2}+\mu^{2}-2\mu \left(c_{i}-o_{i}\right)}{2\sigma^{4}}=0. \end{array}$$ (A.8)

Plugging (A.7) to (A.8), the maximum likelihood estimator of *σ*^2^ is

$$\begin{array}{c} {\hat{\sigma }}_{u}^{2}=\frac{1}{3n}\sum_{i=1}^{n}{\left(2u_{i}-o_{i}-c_{i}\right)}^{2}-\frac{1}{3}{\hat{\mu }}^{2}. \end{array}$$ (A.9)

To verify the solutions are maximum, we show that the Hassian matrix *H* of $\ell (\mu ,\sigma^{2}|\vec{u},\vec{o},\vec{c})$ is negative definite matrix as follows.

$$\begin{array}{c} H=[\begin{array}{cc} \frac{\partial^{2}}{\partial \mu^{2}}\ell & \frac{\partial^{2}}{\partial \mu \partial \sigma^{2}}\ell \\ \frac{\partial^{2}}{\partial \mu \partial \sigma^{2}}\ell & \frac{\partial^{2}}{\partial {\left(\sigma^{2}\right)}^{2}}\ell \end{array}]=[\begin{array}{cc} -\frac{n}{\sigma^{2}} & \frac{n\mu }{\sigma^{4}}-\sum_{i=1}^{n}\frac{\left(c_{i}-o_{i}\right)}{\sigma^{4}}\\ \frac{n\mu }{\sigma^{4}}-\sum_{i=1}^{n}\frac{\left(c_{i}-o_{i}\right)}{\sigma^{4}} & \frac{3n}{2\sigma^{4}}-\sum_{i=1}^{n}\frac{{\left(2u_{i}-o_{i}-c_{i}\right)}^{2}+\mu^{2}-2\mu \left(c_{i}-o_{i}\right)}{\sigma^{6}} \end{array}]. \end{array}$$

Plugging (A.7) and (A.9) to *H*, we obtain

$$\begin{array}{c} H{|}_{\mu =\hat{\mu },\sigma^{2}={\hat{\sigma }}_{u}^{2}}=[\begin{array}{cc} -\frac{n}{{\hat{\sigma }}_{u}^{2}} & 0\\ 0 & -\frac{3n}{2{\hat{\sigma }}_{u}^{2}} \end{array}], \end{array}$$

and it is clear that *H* is a negative definite matrix. The maximum likelihood estimator of (*μ*, *σ*^2^) based on Theorem 2 can be derived analogously and the proof is omitted here.

### Proof of Proposition 2

We derive the one step forecast for the log maximum value. The one step prediction of log minimum value can be obtained by using the same technique. By the joint density of Theorem 1, the marginal distribution of the log maximum variable given the log open variable is

$$\begin{array}{ccc} f(u|o) & = & \int_{-\infty }^{u}\frac{2(2u-o-c)}{\sqrt{2\pi }\sigma^{3}}\;\text{exp}\;\{-\frac{{\left(2u-o-c\right)}^{2}}{2\sigma^{2}}-\frac{\mu^{2}}{2\sigma^{2}}+\frac{\mu (c-o)}{\sigma^{2}}\}dc\\ & = & \text{exp}\;\{\frac{2\mu (u-o)}{\sigma^{2}}\}\int_{-\infty }^{u}\frac{2(2u-o-c)}{\sqrt{2\pi }\sigma^{3}}\;\text{exp}\;\{-\frac{{\left(c-2u+o-\mu \right)}^{2}}{2\sigma^{2}}\}dc\\ & = & \text{exp}\;\{\frac{2\mu (u-o)}{\sigma^{2}}\}\int_{u-o+\mu }^{\infty }\frac{2(x-\mu )}{\sqrt{2\pi }\sigma^{3}}\;\text{exp}\;\{-\frac{x^{2}}{2\sigma^{2}}\}dx\\ & = & \text{exp}\;\{\frac{2\mu (u-o)}{\sigma^{2}}\}[\sqrt{\frac{2}{\pi \sigma^{2}}}\;\text{exp}\;\{-\frac{{\left(u-o+\mu \right)}^{2}}{2\sigma^{2}}\}-\frac{2\mu }{\sigma^{2}}\Phi (-\frac{u-o+\mu }{\sigma })]\\ & = & \sqrt{\frac{2}{\pi \sigma^{2}}}\;\text{exp}\;\{-\frac{{\left(u-o-\mu \right)}^{2}}{2\sigma^{2}}\}-\frac{2\mu }{\sigma^{2}}\Phi (-\frac{u-o+\mu }{\sigma })\;\text{exp}\;\{\frac{2\mu (u-o)}{\sigma^{2}}\},\; \end{array}$$

for *u* > *o*. Then the expectation of *U* given *O* = *o* is

$$\begin{array}{ccc} E[U|O=o] & = & \int_{o}^{\infty }\sqrt{\frac{2}{\pi \sigma^{2}}}ue^{-\frac{{\left(u-o-\mu \right)}^{2}}{2\sigma^{2}}}du-\int_{o}^{\infty }\frac{2\mu u}{\sigma^{2}}\Phi (-\frac{u-o+\mu }{\sigma })e^{\frac{2\mu (u-o)}{\sigma^{2}}}du\\ & = & L_{1}-L_{2}.\;\left(\text{say}\right) \end{array}$$ (A.10)

For the term *L*_1_, we obtain the results by changing the variable.

$$\begin{array}{ccc} L_{1} & = & \int_{o}^{\infty }\sqrt{\frac{2}{\pi \sigma^{2}}}\left(x+o+\mu \right)e^{-\frac{x^{2}}{2\sigma^{2}}}dx\\ & = & \sqrt{\frac{2\sigma^{2}}{\pi }}e^{-\frac{\mu^{2}}{2\sigma^{2}}}+2\left(o+\mu \right)\Phi (\frac{\mu }{\sigma }). \end{array}$$ (A.11)

To tackle the term *L*_2_, we exchange the order of integration as follows.

$$\begin{array}{ccc} L_{2} & = & \int_{o}^{\infty }\frac{2\mu u}{\sigma^{2}}(\int_{u-o+\mu }^{\infty }\frac{1}{\sqrt{2\pi \sigma^{2}}}e^{-\frac{x^{2}}{2\sigma^{2}}}dx)e^{\frac{2\mu (u-o)}{\sigma^{2}}}du\\ & = & \int_{\mu }^{\infty }\frac{1}{\sqrt{2\pi \sigma^{2}}}e^{-\frac{x^{2}}{2\sigma^{2}}}\int_{o}^{x+o-\mu }\frac{2\mu u}{\sigma^{2}}e^{\frac{2\mu (u-o)}{\sigma^{2}}}dudx\\ & = & \int_{\mu }^{\infty }\frac{1}{\sqrt{2\pi \sigma^{2}}}e^{-\frac{x^{2}}{2\sigma^{2}}}[(x+o-\mu -\frac{\sigma^{2}}{2\mu })e^{\frac{2\mu (x-\mu )}{\sigma^{2}}}+(\frac{\sigma^{2}}{2\mu }-o)]dx\\ & = & \int_{\mu }^{\infty }\frac{\left(x+o-\mu -\sigma^{2}/\left(2\mu \right)\right)}{\sqrt{2\pi \sigma^{2}}}e^{-\frac{{\left(x-2\mu \right)}^{2}}{2\sigma^{2}}}dx+(\frac{\sigma^{2}}{2\mu }-o)\int_{\mu }^{\infty }\frac{e^{-\frac{x^{2}}{2\sigma^{2}}}}{\sqrt{2\pi \sigma^{2}}}dx\\ & = & \int_{-\mu }^{\infty }\frac{y}{\sqrt{2\pi \sigma^{2}}}e^{-\frac{y^{2}}{2\sigma^{2}}}dy+(o+\mu -\frac{\sigma^{2}}{2\mu })\int_{-\mu }^{\infty }\frac{e^{-\frac{y^{2}}{2\sigma^{2}}}}{\sqrt{2\pi \sigma^{2}}}dy+(\frac{\sigma^{2}}{2\mu }-o)[1-\Phi (\frac{\mu }{\sigma })]\\ & = & \sqrt{\frac{\sigma^{2}}{2\pi }}e^{-\frac{\mu^{2}}{2\sigma^{2}}}+(o+\mu -\frac{\sigma^{2}}{2\mu })\Phi (\frac{\mu }{\sigma })+(\frac{\sigma^{2}}{2\mu }-o)[1-\Phi (\frac{\mu }{\sigma })]. \end{array}$$ (A.12)

Combining (A.11) and (A.12), the conditional expectation of (A.10) becomes

$$\begin{array}{c} E\left[U|O=o\right]=\sqrt{\frac{2\sigma^{2}}{\pi }}e^{-\frac{\mu^{2}}{2\sigma^{2}}}+o+\mu \Phi (\frac{\mu }{\sigma })-\sqrt{\frac{\sigma^{2}}{2\pi }}e^{-\frac{\mu^{2}}{2\sigma^{2}}}-\frac{\sigma^{2}}{2\mu }[1-2\Phi (\frac{\mu }{\sigma })]. \end{array}$$ (A.13)

Finally, plugging the maximum likelihood estimators of *μ* and *σ*^2^ into (A.13), we claim the results.

### Proof of Corollary 1

By L’Hôpital’s rule, the final term of *U*_*t*_(1) is

$$\begin{array}{c} \lim_{\mu \to 0}\frac{\sigma^{2}\left(1-2\Phi \left(\mu /\sigma \right)\right)}{2\mu }=\lim_{\mu \to 0}\frac{-2\sigma \phi (\mu /\sigma )}{2}=-\frac{\sigma }{\sqrt{2\pi }}. \end{array}$$

Then,

$$\begin{array}{c} \lim_{\mu \to 0}U_{t}\left(1\right)=\sqrt{\frac{2\sigma^{2}}{\pi }}+O_{t+1}-\sqrt{\frac{\sigma^{2}}{2\pi }}+\frac{\sigma }{\sqrt{2\pi }}=O_{t+1}+\sqrt{\frac{2\sigma^{2}}{\pi }}. \end{array}$$

Similar procedure can be applied to *L*_*t*_(1) and we complete this proof.

### Proof of Theorem 4

By Theorem 1, we have the following joint density of (*U*, *C*) conditional on *O* = *o* and $\sigma_{i}^{2}=s$

$$\begin{array}{c} f_{U,C|O,\sigma_{i}^{2}}\left(u,c|o,s\right)=\frac{2(2u-o-c)}{\sqrt{2\pi }s^{3/2}}\;\text{exp}\;\{-\frac{{\left(2u-o-c\right)}^{2}}{2s}-\frac{\mu^{2}}{2s}+\frac{\mu (c-o)}{s}\}. \end{array}$$ (A.14)

Since $\sigma_{i}^{2}$ follows (4), the stationary distribution of $\sigma_{i}^{2}$ is inverse gamma distribution, i.e.,

$$\begin{array}{c} f_{\sigma_{i}^{2}}\left(s\right)=\frac{\beta^{\alpha }}{\Gamma (\alpha )}\;\text{exp}\;\left\{-\beta /s\right\}. \end{array}$$ (A.15)

Then, by using Bayesian method, we obtain the joint density of (*U*, *C*) conditional on *O* = *o* by combining (A.14) and (A.15) as follows.

$$\begin{array}{ccc} f_{U,C|O}\left(u,c|o\right) & = & \int_{0}^{\infty }f_{U,C|O,\sigma_{i}^{2}}\left(u,c|o,s\right)f_{\sigma_{i}^{2}}\left(s\right)ds\\ & = & \frac{2(2u-o-c)}{\sqrt{2\pi }}\frac{\beta^{\alpha }}{\Gamma (\alpha )}\times \\ & & \int_{0}^{\infty }s^{-(\alpha +3/2)-1}\;\text{exp}\;\{-\frac{{\left(2u-c-o\right)}^{2}/2+\mu^{2}/2-\mu \left(c-o\right)+\beta }{s}\}ds\\ & = & \frac{2(2u-o-c)}{\sqrt{2\pi }}\frac{\Gamma \left(\alpha +3/2\right)\beta^{\alpha }2^{\alpha +3/2}}{\Gamma \left(\alpha \right){\left[{\left(2u-c-o\right)}^{2}+\mu^{2}-2\mu \left(c-o\right)+2\beta \right]}^{\alpha +3/2}}. \end{array}$$

Analogously, by using Theorem 2, we can obtain the joint density of (*L*, *C*) conditional on *O* = *o*.

$$\begin{array}{ccc} f_{L,C|O}\left(\ell ,c|o\right) & = & \int_{0}^{\infty }f_{L,C|O,\sigma_{i}^{2}}\left(\ell ,c|o,s\right)f_{\sigma_{i}^{2}}\left(s\right)ds\\ & = & \frac{2(o+c-2\ell )}{\sqrt{2\pi }}\frac{\beta^{\alpha }}{\Gamma (\alpha )}\times \\ & & \int_{0}^{\infty }s^{-(\alpha +3/2)-1}\;\text{exp}\;\{-\frac{{\left(o+c-2\ell \right)}^{2}/2+\mu^{2}/2-\mu \left(c-o\right)+\beta }{s}\}ds\\ & = & \frac{2(o+c-2\ell )}{\sqrt{2\pi }}\frac{\Gamma \left(\alpha +3/2\right)\beta^{\alpha }2^{\alpha +3/2}}{\Gamma \left(\alpha \right){\left[{\left(o+c-2\ell \right)}^{2}+\mu^{2}-2\mu \left(c-o\right)+2\beta \right]}^{\alpha +3/2}}. \end{array}$$

Finally, by using Theorem 3, the joint density of (*U*, *L*, *C*) conditional on *O* = *o* is given below.

$$\begin{array}{ccc} & & f_{U,L,C|O}\left(u,\ell ,c|o\right)=\int_{0}^{\infty }f_{U,L,C|O,\sigma_{i}^{2}}\left(u,\ell ,c|o,s\right)f_{\sigma_{i}^{2}}\left(s\right)ds\\ & = & \sum_{k=-\infty }^{\infty }\frac{4k\left(k+1\right)\beta^{\alpha }}{\sqrt{2\pi }\Gamma \left(\alpha \right)}\times \\ & & \;(\int_{0}^{\infty }s^{-(\alpha +3/2)-1}\;\text{exp}\;\{-\frac{{\left(c+o-2u-2k\left(u-\ell \right)\right)}^{2}/2+\mu^{2}/2-\mu \left(c-o\right)+\beta }{s}\}ds\\ & & \;-{\left(c+o-2u-2k\left(u-\ell \right)\right)}^{2}\times \\ & & \;\int_{0}^{\infty }s^{-(\alpha +5/2)-1}\;\text{exp}\;\{-\frac{{\left(c+o-2u-2k\left(u-\ell \right)\right)}^{2}/2+\mu^{2}/2-\mu \left(c-o\right)+\beta }{s}\}ds)\\ & & -\sum_{k=-\infty }^{\infty }\frac{4k^{2}\beta^{\alpha }}{\sqrt{2\pi }\Gamma \left(\alpha \right)}\times \\ & & \;(\int_{0}^{\infty }s^{-(\alpha +3/2)-1}\;\text{exp}\;\{-\frac{{\left(c-o-2k\left(u-\ell \right)\right)}^{2}/2+\mu^{2}/2-\mu \left(c-o\right)+\beta }{s}\}ds\\ & & \;-{\left(c-o-2k\left(u-\ell \right)\right)}^{2}\times \\ & & \;\int_{0}^{\infty }s^{-(\alpha +5/2)-1}\;\text{exp}\;\{-\frac{{\left(c-o-2k\left(u-\ell \right)\right)}^{2}/2+\mu^{2}/2-\mu \left(c-o\right)+\beta }{s}\}ds)\\ & = & \sum_{k=-\infty }^{\infty }\frac{4k\left(k+1\right)\beta^{\alpha }}{\sqrt{2\pi }\Gamma \left(\alpha \right)}g_{1}-\sum_{k=-\infty }^{\infty }\frac{4k^{2}\beta^{\alpha }}{\sqrt{2\pi }\Gamma \left(\alpha \right)}g_{2}, \end{array}$$

where

$$\begin{array}{ccc} g_{1} & = & \frac{\Gamma \left(\alpha +3/2\right)2^{\alpha +3/2}}{{\left[{\left(c+o-2u-2k\left(u-\ell \right)\right)}^{2}+\mu^{2}/2-\mu \left(c-o\right)+2\beta \right]}^{\alpha +3/2}}\\ & & -\frac{{\left(c+o-2u-2k\left(u-\ell \right)\right)}^{2}\Gamma \left(\alpha +5/2\right)2^{\alpha +5/2}}{{\left[{\left(c+o-2u-2k\left(u-\ell \right)\right)}^{2}+\mu^{2}/2-\mu \left(c-o\right)+2\beta \right]}^{\alpha +5/2}}\\ g_{2} & = & \frac{\Gamma \left(\alpha +3/2\right)2^{\alpha +3/2}}{{\left[{\left(c-o-2k\left(u-\ell \right)\right)}^{2}+\mu^{2}/2-\mu \left(c-o\right)+2\beta \right]}^{\alpha +3/2}}\\ & & -\frac{{\left(c-o-2k\left(u-\ell \right)\right)}^{2}\Gamma \left(\alpha +5/2\right)2^{\alpha +5/2}}{{\left[{\left(c-o-2k\left(u-\ell \right)\right)}^{2}+\mu^{2}/2-\mu \left(c-o\right)+2\beta \right]}^{\alpha +5/2}}, \end{array}$$

which completes the proof.
